# Evaluation of Forgotten Joint Score in total hip arthroplasty with Oxford Hip Score as reference standard

**DOI:** 10.1080/17453674.2019.1599252

**Published:** 2019-04-01

**Authors:** Amanda Larsson, Ola Rolfson, Johan Kärrholm

**Affiliations:** a Department of Orthopaedics, Institute of Clinical Sciences, Sahlgrenska Academy, University of Gothenburg, Gothenburg;;; b The Swedish Hip Arthroplasty Register, Registercentrum, Västra Götaland, Gothenburg, Sweden

## Abstract

Background and purpose — Total hip arthroplasty (THA) is performed mainly because of pain. To evaluate the result after surgery, different questionnaires measuring the patient-reported outcome regarding quality of life are used. Forgotten Joint Score (FJS), designed to chart postoperative symptoms, was developed to find subtle differences between patients who report that their operated hip is “very good” or “excellent.” We evaluated whether FJS provides additional information compared with the Oxford Hip Score (OHS) and ceiling and floor effects with use of these instruments. We also studied level of internal consistency for OHS and FJS, and the reproducibility of the FJS.

Patients and methods — 111 patients who underwent unilateral primary THA in 2015 were included. The participants answered 2 questionnaires: Forgotten Joint Score and Oxford Hip Score. Floor and ceiling effects were recorded for each of the instruments and agreement between them. The FJS was studied with respect to reproducibility and level of internal consistency.

Results — OHS ceiling effect (31%) was higher compared with FJS (21%), whereas the OHS seemed to provide a more nuanced picture of patients with an inferior clinical result. Floor effect for FJS was 3% and 0% for OHS. The degree of explanation was 68% between the 2 questionnaires (linear regression, r^2^ = 0.68). FJS items had a high internal consistency (Cronbach’s a = 0.93) and reproducibility (Pearson correlation = 0.87, ICC = 0.93); 92 patients answered on 2 distributions of the FJS questionnaires, 19 patients had identical answers.

Interpretation — OHS had a larger ceiling effect than FJS, which could indicate that FJS is a more fine-tuned instrument to separate patients with good to excellent outcome after THA. The high internal consistency of FJS indicates that the items of the instrument consistently cover the construct of joint awareness.

The aim with total hip arthroplasty (THA) is to relieve pain, and improve joint mobility, physical ability, and quality of life. Since patients’ expectations on the postoperative pain and functional outcomes have changed over the past 20 years (Hamilton et al. 2017), it is of importance to apply validated methods to measure patient-reported outcomes (PROs) after surgery (Behrend et al. 2012). Questionnaires measuring PROs should preferably exhibit low ceiling and floor effects. Critical ceiling and floor effects are regarded to be present if 15% or more of the population reach the maximum or minimum score of a scale, respectively (Terwee et al. 2007). Ceiling and floor effects could make it difficult to study the development over time, since the true results and changes at follow up are concealed.

The commonly used Oxford Hip Score (OHS) and the more recently developed Forgotten Joint Score (FJS) are 2 validated metrics for the evaluation of THA. OHS focuses on the preoperative status, while FJS primarily was designed to chart the symptoms postoperatively (Hamilton et al. 2016). Wylde et al. (2005) explored different weaknesses of the OHS questionnaire, e.g., that patients experienced that some questions did not have a clear meaning. The patients also commented on the difficulty of answering according to their “average pain” during the past 4 weeks, since their pain sometimes fluctuated based on current medication and level of physical activity. Some of the questions in OHS are so called “double-barreled questions,” meaning there is more than 1 claim in each question. This could result in difficulty in interpreting the answers, since some patients marked more than 1 of the possible answers for each question. In addition, the OHS has been criticized for exhibiting ceiling effects at postoperative follow-ups (Hamilton et al. 2016, 2017). The FJS was developed in 2012 as a reaction to the shortcomings of established measures following joint replacement and its use is growing.

The Swedish Hip Arthroplasty Register established a program collecting PROs in 2002 (Rolfson et al. 2011). The Registry direction is currently investigating new or alternative measures to include in the program. As both the newly developed FJS and the well-established OHS are tentative candidates, we sought to investigate these measures in a typical THA population. Hence, the objective of this study was to evaluate whether Forgotten Joint Score (FJS) provides more information compared with, or in addition to, Oxford Hip Score (OHS) regarding the clinical outcome after THA. Specifically, we aimed to compare ceiling and floor effects between OHS and FJS, to investigate the level of internal consistency for OHS and FJS, and to test the reproducibility of the FJS.

## Patients and methods

### Study population (Figure 1)

200 patients who had undergone unilateral THA at the Department of Orthopaedics, Sahlgrenska University Hospital in Mölndal during 2015 were invited to participate. The population was selected from a consecutive series of THA with stratification for age and sex: half of the invited patients were over 65 years old and the other half were 65 years old or younger; half of the invited patients in each age group were females. All types of diagnoses (Table 1) and patients who previously had been operated on their opposite hip were included in the study. 1 of the 200 patients had been revised at the time when the questionnaires were sent out. This patient was also included.

**Table 1.** Patient demographics: ASA classification and Charnley category in 200 patients (200 hips) primarily selected to be invited

	All n = 200	Age < 65 n = 99	Age ≥ 65 n = 101
Male/female	100/100	50/49	50/51
Age, median	66	57	75
range	17–97	17–64	66–97
Diagnosis			
Primary osteoarthritis	135	66	69
Inflammatory joint disease	1	1	0
Fracture	36	10	26
Sequelae childhood hip disease	10	10	0
Femoral head necrosis	18	12	6
Charnley category^a^			
A	84	44	40
B	79	32	8
C	18	10	47
missing	19	13	6
ASA			
1	49	39	10
2	118	49	69
3	30	9	21
4–5	0	0	0
missing	3	2	1

a Filled in by patient 1 year after the index operation.

### Sample and logistics

Invited patients were asked to complete 2 questionnaires, FJS and OHS, which were sent out by ordinary mail at the beginning of September 2017. Questionnaires were filled in at least 1 year after the primary index operation. 10 to 14 days after return of the questionnaire, the FJS was sent out once again to evaluate its reproducibility. Approximately 1 month after the first invitation to participate was sent out, patients who had not responded received a phone call reminder and an offer to receive a new set of questionnaires. If the patient declined participation or did not answer after 2 phone calls no further attempts to reach the patients were made. Numbers included were calculated based on an estimated response rate of 75%, i.e., 150 patients.

### Patient-reported outcome measures

#### Oxford Hip Score

The OHS, developed in 1996 (Dawson et al. 1996), is a patient-centered, 12 item-questionnaire with questions concerning pain and physical ability that the patient experienced during the past 4 weeks. The OHS originally used a scoring system ranging between 1 and 5 (worst–best). Since 2007, OHS items range from 0 to 4 where 4 is the best, which leads to a total score ranging from 0 to 48, where 48 equals the best outcome (Murray et al. 2007). When interpreting the answers and calculating the overall score of OHS, a maximum of 2 missing values are accepted. If the patient fills in more than 1 answer per question, the worst response should be used when calculating the total score (Nilsdotter and Bremander 2011).

#### Forgotten Joint Score

The Forgotten Joint Score (FJS) is a joint-specific questionnaire developed in 2012 (Behrend et al. 2012) with the aim to measure PRO of joint disorders (Hamilton et al. 2017). FJS is designed to measure the ability of the patient to “forget” about their problematic joint after treatment. FJS is available in 3 versions: hip, knee, and shoulder. Studies imply that older questionnaires do not provide quite as variegated a picture of the results, as they mostly differ between “good” and “bad.” Behrend et al. (2012), however, states that since FJS differ between “good,” “very good,” and “excellent” on a 5-grade Likert scale ranging from “never” to “mostly,” it could reduce the risk of ceiling effects. As opposed to, for example OHS, FJS is a questionnaire that focus on the awareness, instead of the pain, of the affected joint (Hamilton et al. 2017). 4 missing values are regarded as acceptable when the scores are summarized and transformed to a scale ranging from 0 to 100, where a high value indicate that the patient tends to be less aware of the affected joint when performing daily activities (Behrend et al. 2012). According to the official website of FJS (http://www.forgotten-joint-score.info/), the developers have undertaken translation into several languages (including Swedish) and subsequently linguistically validated the translated forms based on the Principles of Good Practice for the Translation and Cultural Adaption Process for Patient-Reported Outcomes (PRO) Measures (Wild et al. 2005).

### Reasons for non-participation

Of those patients receiving a reminder phone call, 30 did not answer. A further 16 patients accepted to participate in the study, but did not, despite their positive answer, return any questionnaires (Table 2, see Supplementary data).

### Ceiling and floor effect

A critical ceiling or floor effect was regarded to be present if 15% or more of the patients chose the best or worst possible answer when answering a specific question (Terwee et al. 2007). Ceiling or floor effects at or above this level indicate limited content validity and reduced reliability.

### Statistics

Cronbach’s a was used to measure the internal consistency among the 7 questions that were assessed to measure the same health dimension in the 2 questionnaires. The relationship between total OHS and FJS was assessed using simple linear regression. The intraclass correlation coefficient (ICC) and Pearson’s correlation were calculated to evaluate the reproducibility of the questionnaires. Statistical analyses were performed using IBM SPSS Statistics version 24 (IBM Corp, Armonk, NY, USA).

### Ethics, funding, and potential conflicts of interest

The study was approved by the Regional Ethical Review Board in Gothenburg (ref 607–17). OR and JK were financed by grants from the Swedish state under the agreement between the Swedish government and the county councils, the ALF agreement (ALFGBG- 522591 and 721791, respectively). The authors have no conflict of interest.

## Results

### Ceiling and floor effects of FJS and OHS

Of the 199 patients primarily invited, 111 (56%) answered the questionnaires. Of these, 52% were female. The median age was 69 (19–100). Mean OHS was 42 (SD 9, median 45, IQR 40–48) and mean FJS was 64 (SD 32, median 71, IQR 42–93). 68% to 94% chose option 1 (best) for each of the questions in the OHS questionnaire. The corresponding proportion for FJS ranged from 39% to 77%. The responses on the FJS forms were more scattered among the different options, and all response options were chosen for all questions, which differs from OHS, where no patients chose option 5 (worst) on 3 questions (questions 2, 3, and 4. Tables 3 and 4, see Supplementary data). The total ceiling effect for OHS was 31% and FJS 21%. The floor effect did not reach 15% for either questionnaire, but was slightly higher for FJS (FJS 3%, OHS 0%).

### Level of internal consistency (Cronbach’s a) and agreement between questionnaires

113 patients responded to the first distribution of FJS (Cronbach’s a = 0.97) and 111 patients answered the OHS (Cronbach’s a = 0.93). In the linear regression analysis, the degree of explanation between the 2 instruments reached 68% (r^2^ = 0.68, Figure 2). The mean difference between FJS and OHS was 22, i.e., FJS was mean 22 lower than OHS when converted to a 0–100 scale (Bland–Altman limits of agreement, Figure 3).

**Figure 1.**
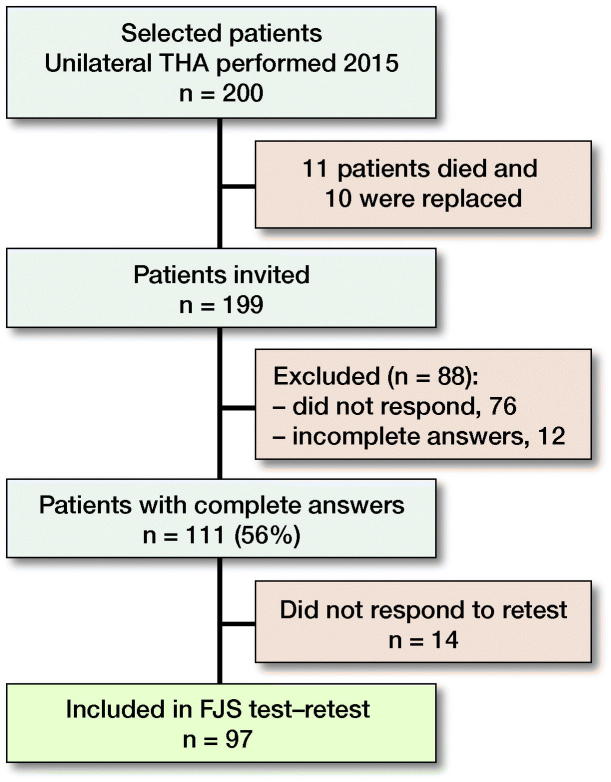
Participation.

**Figure 2.**
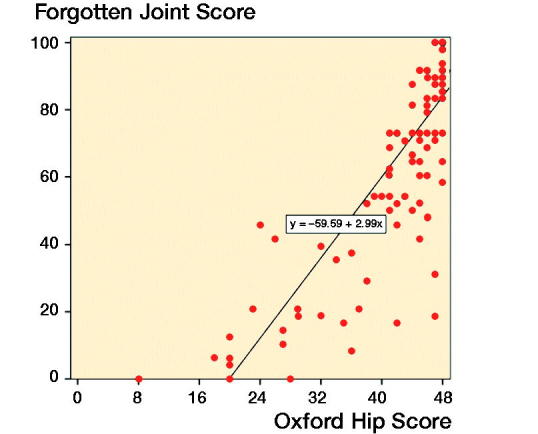
Linear regression analysis. The degree of explanation between the 2 instruments reached 0.68.

**Figure 3.**
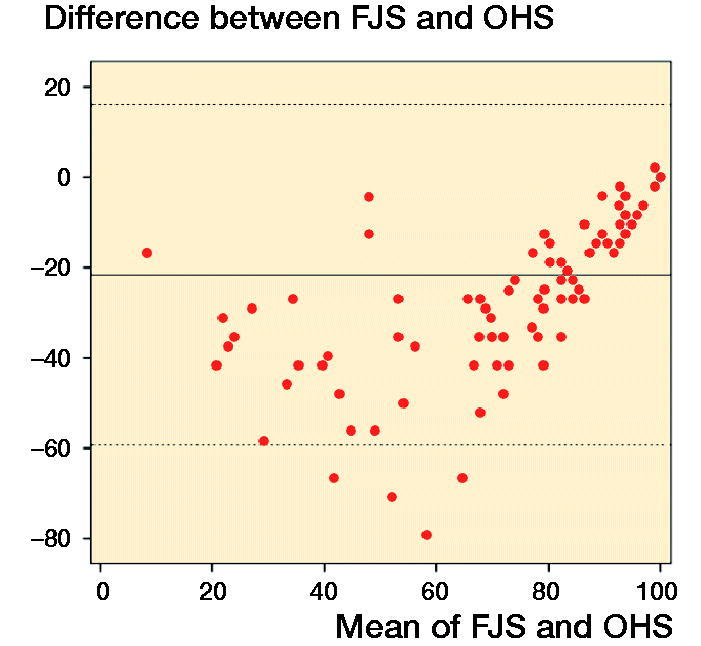
Bland–Altman limits of agreement.

### Forgotten Joint Score reproducibility

The Pearson correlation coefficient and the intraclass correlation (ICC) for the reproducibility of the FJS were 0.87 (95% CI 0.75–0.96) and 0.93 (95% CI 0.89–0.95).

## Discussion

This pilot study emanated from the Swedish Hip Arthroplasty Register to explore and evaluate new instruments with potential use for specific or general purposes in the follow-up after THA. We found that the FJS had an acceptable and good reproducibility. It also had less of a ceiling effect when compared with the OHS. Because of these properties, the FJS could be considered for many purposes and especially in the evaluation of new implants claimed to provide improved patient satisfaction and/or clinical performance.

### Ceiling and floor effects of FJS and OHS

Studies have shown that OHS has a risk for ceiling effects postoperatively (Hamilton et al. 2016, 2017), which also was found by us. These ceiling effects were greater for OHS for each of the 7 correlating questions as well as the overall ceiling effect, which further support our hypothesis that FJS is a more nuanced instrument when measuring the results after THA.

According to Terwee et al. (2007), a ceiling effect is present if more than 15% of the participants score the “best” result, corresponding to the lowest possible score for OHS and FJS. In a study of patients operated with either total knee or total hip arthroplasty, Hamilton et al. (2016) found a ceiling effect in the latter group of 8% at 6 months, which increased to 10% at 1 year, i.e., well below this limit (Hamilton et al. 2016). In our study, the ceiling effect for the OHS and FJS were higher at 31% and 21% respectively. The reason for this is not known, but longer follow-up could have contributed. Neither of the 2 questionnaires reached 15% for floor effect, though 3% achieved the maximum score on FJS. Several patients achieved high total FJS values, which indicates high awareness of their hip joint in daily life. Thus, the FJS provides a better differentiation of hip-related symptoms in patients who are generally satisfied with their THA operation. So far, the majority of studies which have compared the FJS with other scoring systems, have found that the ceiling effect of FJS is smaller or about the same as the reference used. Matsumoto et al. (2015) compared FJS with the WOMAC and the Japanese Orthopaedic Association Hip Disease Evaluation Questionnaire (JHEQ) in 108 patients operated with THA about 2.5 years after the operation. A low ceiling effect was reported for the FJS (4%) and for the JHEQ (3%), (Matsumoto et al. 2015).

Hamilton et al. (2016) suggested that FJS is more responsive to change than OHS based on findings of a more pronounced change in the FJS than for the OHS between the 6- and 12-month follow-up. They also noticed that the measured ceiling effect was nearly doubled for OHS compared with FJS (21% and 10% respectively). In another study by Hamilton et al. (2017), they found a pronounced floor effect for FJS when used preoperatively. 22% of the THA patients achieved the lowest score. These number differs from OHS, where no floor effects were shown preoperatively. The ceiling effect, however, was approximately half for FJS 1 year postoperatively compared with OHS.

### Reproducibility and internal consistency

To examine the reproducibility of FJS, a comparison was made between the answers of the 1st and 2nd distribution of FJS. Overall, patients chose the same response option for both questionnaires in the test–retest analysis. Fewer patients, however, answered on the second distribution of FJS (n = 92). 19 patients had identical answers on both FJS 1 and FJS 2 and the internal consistency was high (ICC = 0.93).

Thomsen et al. (2016) found good reliability in test–retest of FJS total score (ICC = 0.91). When FJS was compared with the Oxford Knee Score (OKS), there was a high level of internal consistency (Cronbach’s a = 0.96). Behrend et al. (2012) also observed that FJS had high internal consistency (Cronbach’s a = 0.95). Both these studies and ours indicate that the items of FJS consistently measure the construct of joint awareness.

### Weaknesses of this study

Our study was performed in a Swedish population. To what extent this circumstance has influenced our result is not known, but we think that the influence of differences in ethnicity between nations or groups is weak, at least in Europe.

Since this was a cross-sectional study, change over time was not analyzed, which is a limitation of this study, as well as the fact that only primary THA patients were included. The response frequency was lower compared with the Register’s ordinary PROM routine (Rolfson 2016), which could be due to a number of extra questionnaires with stated research purpose outside normal routines.

Another possible limitation of this study was that a moderately sized population was included. The calculated participation level was 75%, i.e., 150 respondents, and the actual participation level was 56%. 123 of the patients answered the FJS completely and 111 patients answered the OHS completely. The response frequency probably would have been higher if the study took place over a longer period and more attempts could have been made to reach the patients who did not answer.

Despite the fact that the FJS had a lower ceiling effect than the OHS, 1 of 5 nevertheless reached the maximum score for all items included. This finding raises the question whether still more sensitive instruments should be used, especially in situations when implants used in surgical procedures supposed to address high-demand patients are operated. It could be that new instruments used to evaluate patients with femoroacetabular impingement have still less ceiling effect in an arthroplasty population than the FJS (Thorborg et al. 2011, Griffin et al. 2012, Mohtadi et al. 2012). These questionnaires do, however, include items related to high-demand sport and work-related activities, these not being relevant for a majority of the population operated with total hip arthroplasty. Thus, these instruments might be less suitable to use in arthroplasty registers.

### Conclusion

We found that FJS could be used as an alternative to OHS in primary THA. The answers on FJS are more scattered than on OHS, which could indicate that FJS provides a more variegated picture of the clinical results in this population. The ceiling effect of FJS was lower compared with that of OHS, which provides valuable information, not least in a field where new implants with proposed superior performance are continuously introduced and patient expectations in terms of the results tend to increase. On the other hand, FJS might, due to its floor effect, be less suitable to predict the need for future revision surgery. Further studies in the Swedish population of patients with THA are needed, preferably with a larger study population, to establish whether the Forgotten Joint Score is sensitive to change and if it could be included in the Swedish Hip Arthroplasty Register PROMs routine with maintained completeness.

### Supplementary data

Tables 2–4 are available as supplementary data in the online version of this article, http://dx.doi.org/10.1080/17453674.2019.1599252

JK conceived and planned the study. AL managed questionnaire logistics. AL and OR performed the statistical analyses. AL drafted the manuscript. All authors interpreted the results.

*Acta* thanks Ove Furnes for help with peer review of this study.

## Supplementary Material

Supplemental Material

## Supplementary data

Tables 2-4 are available as supplementary data in the online version of this article, http://dx.doi.org/10.1080/17453674.2019.1599252
