# Supplementary material for: Evaluation of Forgotten Joint Score in total hip arthroplasty with Oxford Hip Score as reference standard
Source: Acta Orthop. 2019 Apr 1;90(3):253–7. doi: 10.1080/17453674.2019.1599252 (PMC6534202; doi:10.1080/17453674.2019.1599252)
Supplement: Supplemental Material [file IORT_A_1599252_SM6420.pdf]

Supplementary data

Table 2. Reasons for non-participation given by patients when receiving a reminder phone call

Reason for non-participation	Number of patients
Not answering phone call	30
Missing valid phone number	7
Recent illness/hospitalized	5
On vacation	1
Dissatisfied with the surgery	2
Not interested	16
Accepted participation, but did not return questionnaires	15
Total	76

Table 3. Forgotten Joint Score: distribution of answers for each question

	FJS answer					
Question no.	1	2	3	4	5	Total
1	58	33	2	14	10	117
2	53	20	10	19	15	117
3	57	13	9	22	16	117
4	77	13	8	11	9	118
5	59	18	13	20	6	116
6	50	25	9	14	20	118
7	43	19	15	23	17	117
8	40	22	12	23	21	118
9	42	21	18	20	17	118
10	41	24	19	20	14	118
11	42	16	11	26	20	115
12	39	19	11	11	18	117

The discrepancy in total numbers is explained by incompletely answered questionnaires by some patients.

Table 4. Oxford Hip Score: distribution of answers for each question

	OHS answer					
Question no.	0	1	2	3	4	Total
1	74	28	9	3	4	118
2	90	18	10	1		119
3	78	18	19	4		119
4	68	34	9	8		119
5	86	22	8	1	1	118
6	80	12	10	5	8	115
7	72	28	8	9	1	118
8	87	13	10	1	6	117
9	71	25	3	8	10	117
10	86	10	12	6	4	118
11	81	13	14	7	3	118
12	94	5	11	2	6	118

The discrepancy in total numbers is explained by incompletely answered questionnaires by some patients.
